# Entropy-Driven Phase Separation of AIE Polysiloxanes into Porous Fibrous Films for Fluorescence Sensing

**DOI:** 10.3390/polym17243252

**Published:** 2025-12-06

**Authors:** Jingxuan Zhu, Ruirui Shi, Yifan Wang, Yan Chen, Yan Liang, Hua Wang, Chuanjian Zhou

**Affiliations:** 1 School of Materials Science and Engineering, Shandong University, Jinan 250061, China; 202434207@mail.sdu.edu.cn (J.Z.); rruishi@mail.sdu.edu.cn (R.S.); wangyf_wx@163.com (Y.W.); 202334179@mail.sdu.edu.cn (Y.C.); zhouchuanjian@sdu.edu.cn (C.Z.); 2 State Key Laboratory of Coatings for Advanced Equipment, Jinan 250061, China; 3 Key Laboratory of Special Functional Aggregated Materials, Ministry of Education, Jinan 250100, China; 4 School of Food Science and Technology, Qilu University of Technology, Jinan 250353, China; 5 Weihai Research Institute of Industrial Technology, Shandong University, Weihai 264209, China

**Keywords:** phase separation, island-in-the-sea structure, molecular dynamics simulations

## Abstract

Translating the exceptional luminescent properties of AIEgens into efficient and practical sensing devices has long been a major challenge restricting their practical application. In this work, we demonstrate a novel strategy based on phase separation to fabricate stable, high-surface-area sensing films that address the fluorescence quenching typically associated with conventional nanospheres. Fluorescent polysiloxanes bearing tetraphenylphenyl (TPP) side groups were synthesized and processed into fibrous films via electrospinning. Leveraging the intrinsic incompatibility of the polymer, entropy-driven phase separation generated an “sea–island” morphology. This hierarchical structure significantly enlarged the specific surface area and facilitated analyte diffusion, thereby improving the accessibility of active sites. Molecular dynamics simulations not only predicted the formation of this architecture but also clarified the underlying entropy-driven mechanism. Overall, this work provides a solid foundation and conceptual framework for investigating how quantitative regulation of lumogenic unit density and spatial distribution governs sensing performance.

## 1. Introduction

Fluorescent polymers have demonstrated great potential for sensing applications in drug delivery [1,2], fluorescent bioprobes [3,4,5,6], and optoelectronic devices [7,8], especially in fluorescent sensing applications [9,10,11,12,13,14]. The aggregation-caused quenching (ACQ) of conventional fluorophores was resolved by the 2001 introduction of aggregation-induced emission (AIE) [15], a breakthrough that accelerated the development of various AIE materials [16,17]. In recent years, AIE-active polymers, particularly siloxane-based AIE systems, have attracted increasing attention owing to their high flexibility, excellent processability, and tunable photophysical behaviors [18,19]. Polysiloxane-based AIE materials bearing aromatic rotors can generate strong emission through restriction of intramolecular motion (RIM), but most existing systems rely on nanoparticles or cast films, where aggregation is kinetically constrained and interfacial accessibility is limited [20,21]. Therefore, translating the luminescent properties of AIEgens into efficient and practical sensing devices remains a critical challenge [22,23]. To bridge this gap, we propose a paradigm shift from using AIEgens as additives to constructing them as inherent functional components. The developed TPP-functionalized polysiloxanes embody this “material-as-probe” concept, enabling the creation of structured sensing interfaces with optimized molecular accessibility and operational stability.

In our previous work, a tetraphenylbenzene (TPB)-based derivative exhibited aggregation-induced emission enhancement (AIEE) at submicromolar concentrations, a phenomenon termed the ‘silicon core effect’ [24,25,26,27]. Building on this, we developed TPB-modified polysiloxanes for detecting picric acid [28]. However, the resulting porous nanoparticles still suffered from solid-state aggregation akin to ACQ, which quenched fluorescence and limited sensing performance by reducing the accessible surface area. To overcome this limitation and enhance interfacial responsiveness, we herein demonstrate a one-step electrospinning strategy to fabricate a “sea–island” structured fibrous film using AIEE phenyl-modified polysiloxanes. To the best of our knowledge, this work represents the first realization of sea–island architectures formed by AIE-functionalized polysiloxane molecular systems within electrospun fibrous films. This concept is fundamentally distinct from conventional AIE nanospheres or cast-film systems, as the rapid solvent evaporation and composition-dependent phase separation during electrospinning cooperatively drive the formation of well-defined dispersed TPP-rich “islands” embedded in a polysiloxane “sea”.

During the spinning process, the incompatibility between the fluorescent polymer and the fiber matrix leads to phase separation [29]. Meanwhile, driven by entropy-driven, the fluorescent polymer spontaneously self-assembles into porous nanoparticles. As a result, a uniformly distributed sea–island microstructure is established within the fiber matrix. Compared with conventional smooth films, this island-like morphology markedly increased the specific surface area of the sensing interface, thereby amplifying the number of active TPB moieties available for interaction with target analytes per unit area. Guided by molecular dynamics simulations, we confirmed that this structure originated from polymer phase separation [30,31,32,33] thus providing theoretical support for the feasibility of our approach. Establishing such sensing architectures is crucial for optimizing material responsiveness and signal output, and provides a fundamental framework for quantitatively modulating fluorophore density [34]. This work offers novel insights and methodologies for the practical application of AIE-based fluorescent polymer sensors.

## 2. Materials and Methods

### 2.1. Materials

Octamethylcyclotetrasiloxane (D_4_, 98%, Bidepharm, Shanghai, China), 2,4,6,8-Tetramethyl-2,4,6,8-tetravinylcyclotetrasiloxane (D_4_Vi, 98%, Bidepharm), 1,3-Diethyl-1,1,3,3-Tetramethyl Disiloxane (95%, Macklin, Shanghai, China), Diphenyl oxide (99%, Bidepharm), Dibenzyl ketone (97%, Bidepharm), Benzil (99%, Adamas, Shanghai, China).

### 2.2. Characterization

Fourier transform infrared (FTIR) spectra were recorded on a TENSOR 37 spectrometer (Bruker Optik GmbH, Ettlingen, Germany) in the range of 4000–400 cm^−1^. Nuclear magnetic resonance (NMR) spectra, including ^1^H and ^13^C, were obtained using a Bruker AVANCE 400 spectrometer with CDCl_3_ as the solvent. The intrinsic viscosity ([η]) of methylvinylsiloxane (MVQ) samples with varying vinyl group contents was measured using an Ubbelohde viscometer, and the viscosity-average molecular weight (M_η_) was calculated using the Mark–Houwink equation [η] = KM^*α*^, where K = 0.3 and *α* = 0.62. Fluorescence emission and ultraviolet-visible (UV-vis) absorption spectra were recorded on an RF-6000 spectrofluorophotometer and a UV-2600 spectrophotometer (SHIMADZU, Kyoto, Japan), respectively. To study AIE behavior, polymer solutions in tetrahydrofuran (THF) were prepared at defined concentrations and gradually introduced into deionized water under vigorous stirring.

Fluorescence lifetime measurements were performed on an FLS920 spectrometer (Edinburgh Instruments, Livingston, UK) equipped with an EPL picosecond pulsed light source, with excitation at 370 nm and emission monitored at 450 nm. The instrument provides a minimum lifetime measurement range of 50 ps, time resolution at the picosecond scale, and emission detection across 200–1400 nm with a wavelength accuracy of 0.01 nm and a signal-to-noise ratio of 1:6000. The samples consisted of aqueous solutions containing 1 µM TPP-MQs nanoparticles, prepared as described in the previous section.

Thermogravimetric analysis (TGA) was conducted on a Labsys Evo TGA/DSC thermal analyzer (Setaram, Lyon, France). The experiments were carried out under an argon atmosphere with a temperature ramp from 303 K to 1073 K at a heating rate of 20 K·min^−1^.

### 2.3. Synthesis of Methylvinyl Polysiloxane (MVQ)

**Scheme 1:**
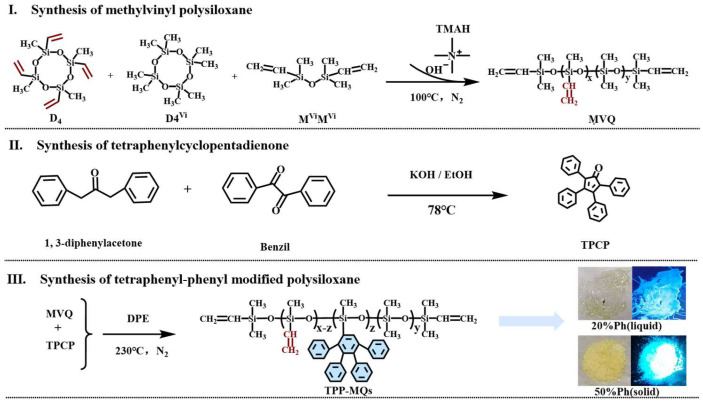
Synthesis of TPP-MQs and the phase change in samples as the content of TPP groups increases.

The synthesis processes are shown in Scheme 1. The synthesis of MVQ is achieved through the ring-opening reaction of cyclotetrasiloxane (D_4_). Equal molar amounts of D_4_ and D_4_^Vi^ were mixed, and tetramethylammonium hydroxide (TMAH) was added as a catalyst. The mixture was then heated under reduced pressure at 50 °C for 1 h to remove water. Subsequently, 1,3-Diethenyl-1,1,3,3-tetramethyldisiloxane (M^Vi^M^Vi^) was introduced as an end-capping agent. The reaction mixture was heated to 100 °C and maintained for 3–4 h until the system viscosity stabilized. Finally, the temperature was gradually increased to 150 °C under reduced pressure to remove low-boiling-point substances and decompose the catalyst. After cooling the system to room temperature, the target product was isolated by extraction and filtration. The vinyl group content in the silicone oil can be tailored by adjusting the feed ratio of D_4_ to D_4_^Vi^ .

FT-IR (cm^−1^): 3055, 3018, 2962, 1597, 1407, 1259, 1073, 1010, 958, 786, 684. ^1^H NMR (CDCl_3_, 300 MHz, ppm): δ 5.2–6.2 (t, Si-CH=CH_2_), −0.2–0.4 (t, Si-CH_3_).

### 2.4. Synthesis of Tetraphenylcyclopentadienone (TPCP)

1, 3-diphenylacetone and benzil were dissolved in an adequate quantity of ethanol and heated to 78 °C. Potassium hydroxide–ethanol solution was then added dropwise to the system, causing a rapid color change from light yellow to dark purple. The reaction was allowed to proceed for 15 min after the complete addition of the reagent, followed by cooling the system to room temperature. After extraction and filtration, the product was washed twice with ethanol and then dried under a vacuum oven, yielding the product (TPCP) with a yield exceeding 90%.

^13^C NMR (CDCl_3_, 300 MHz, ppm): 125.36, 127.52, 128.05, 128.09, 128.56, 129.39, 130.20, 130.80, 133.11, 154.53, 200.38.

### 2.5. Synthesis of Tetraphenyl-Phenyl Modified Polysiloxane (TPP-MQs)

The reaction system, consisting of MVQ and a slight excess of TPCP (quantities specified in Table S1), was dissolved in 20 mL of diphenyl ether as the solvent. The mixture was maintained at 230 °C for 8–10 h, during which the disappearance of the purple coloration indicated the completion of the reaction. Subsequently, a large volume of methanol was added to the cooled reaction mixture, which was allowed to stand for 30 min to facilitate precipitation. The resulting precipitate was separated using a separatory funnel, then dissolved in dichloromethane. This purification cycle—precipitation with methanol, isolation, and dissolution in dichloromethane—was repeated 2–3 times until pure yellow silicone oil was obtained through washing. Finally, the product was dried in a vacuum oven overnight to yield purified TPP-MQs. The TPP grafting rates for each sample are summarized in Table S2.

### 2.6. Molecular Dynamics Simulations

Initial molecular dynamics models were constructed using the Amorphous Cell module in Materials Studio 2022 under the COMPASS force field, with periodic boundary conditions applied [35]. The assembly protocol for amorphous cells containing varying additive concentrations is illustrated. Initially, TPP-MQs polymer chains were randomly copolymerized according to experimental specifications, comprising 14 dimethyl units and 6 methylphenyl units. Polycaprolactone (PCL) molecules were modeled with 50 caprolactone repeating units. Before the composite system assembly, polymer chains underwent geometry optimization using the Smart algorithm, with convergence thresholds set at 1 × 10^−3^ kcal/mol/Å for forces and 2 × 10^−5^ kcal/mol for energy. Periodic simulation cells were then generated, containing 2 PCL chains and 3 TPP-MQs chains (mass ratio ≈ 1:2). For each configuration, 5 independent models were randomly created using the Amorphous Cell module and subjected to rigorous geometry relaxation. Low-energy configurations were selected for thermal annealing (0.1 MPa, 300–500 K, 2 ns) to alleviate local energy minima. Final structural equilibration involved: (1) a 1 ns NPT simulation at 0.1 MPa, followed by (2) a 500 ps NVT simulation at 298 K. Data acquisition was performed using the final 100 ps trajectory from NVT production runs.

## 3. Results

The Diels–Alder reaction serves as a rapid and efficient synthetic strategy for constructing AIE-active polymer [26,28]. Comprehensive structural analyses of the precursor MVQs and the resultant TPP-MQs, as detailed in the Supporting Information, confirm successful synthesis. Fundamental material properties were systematically evaluated through ^1^H NMR spectral analysis, with critical parameters summarized in Table S2. Samples containing less than 30% TPP groups retain liquid-phase characteristics, with viscosity enhancements proportional to TPP concentration. However, beyond the 30% TPP threshold, intensified intermolecular interactions effectively suppress molecular mobility, leading to solid-state formation at ambient temperature (as shown in Scheme 1(III)). Particularly noteworthy is the system with a 65% grafting ratio, where substantial steric hindrance from TPP substituents induces pronounced rotational constraint effects on polymer chain segments.

**Figure 1:**
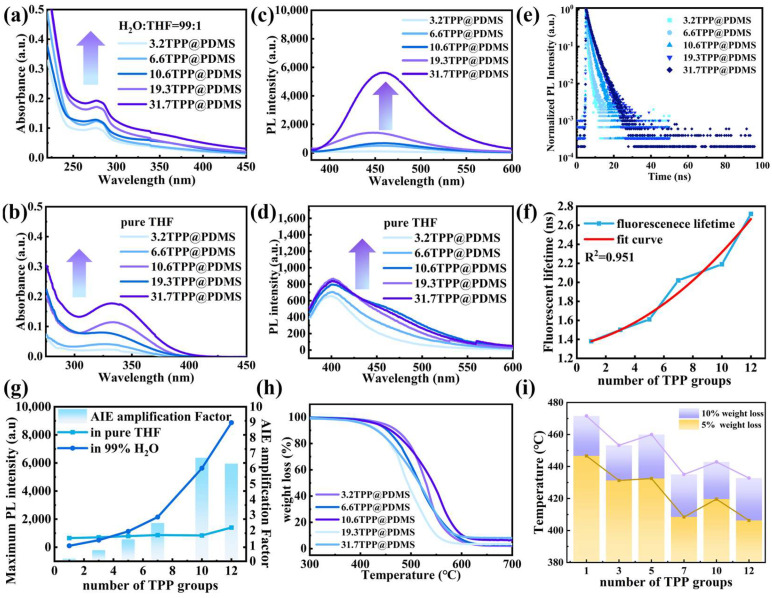
(**a**–**d**) UV-vis absorption and fluorescence spectra (λex = 330 nm) of 1 M TPP-MQs in H_2_O/THF (99:1 *v*/*v*) solution and in pure THF. (**e**) Normalized time-resolved fluorescence decay curves of TPP-MQs with varying TPP content. (**f**) Average fluorescence lifetime (τ) as a function of the number of TPP groups per polymer chain (the red line represents an exponential fit to the lifetime trend). (**g**) AIE properties of TPP-MQs. (**h**) Thermogravimetric (TG) curve of TPP-MQs. (**i**) Temperatures corresponding to 5% and 10% weight loss.

The UV-vis absorption spectra of TPP-MQs under two different environments are presented in Figure 1a,b. The sample nomenclature (e.g., 3.2TPP@PDMS) denotes the molar percentage (mol%) of TPP units grafted onto the PDMS backbone, which is pivotal for understanding the subsequent photophysical properties. In THF solution, the absorption spectrum exhibits a characteristic maximum at 330 nm, while in aqueous systems, the absorption peak appears relatively smooth and shifts below 300 nm attributable to π-π stacking-induced molecular aggregation. Additionally, an increase in the content of TPP groups within the polymer molecules results in a gradual enhancement of UV-vis absorption intensity, confirming that the absorption intensity is significantly influenced by the TPP content.

The fluorescence spectra of TPP-MQs in pure THF (soluble) and 99% H_2_O (insoluble) are depicted in Figure 1c,d respectively. In the H_2_O/THF mixed solvent system, an increase in the TPP group content significantly enhanced the fluorescence intensity, demonstrating distinct aggregation-induced emission (AIE) characteristics. In contrast, in a pure THF solution, the effect of increasing TPP group content on fluorescence intensity was minimal. This can be attributed to the fact that, in pure THF, the polymer solution behaves as a dilute solution of TPP groups, where small variations in TPP content have negligible effects on fluorescence intensity. However, in a 99% H_2_O system, the polymer separates into liquid droplets and exists in an aggregated form as shown in Figure 1g. To quantitatively evaluate the AIE efficiency, Figure 1g presents the amplification factor of the fluorescence intensity, defined as the ratio (I_a_gg/I_s_ol) of the intensity in the aggregated state (99% H_2_O) to that in the solution state (pure THF). It is evident that polymers with higher TPP group content tend to have a greater concentration within the droplets, resulting in more pronounced restriction of intramolecular rotation. This leads to an enhanced AIE property. This observation underscores the significant influence of the intermolecular distance among TPP groups on the AIE property. As the TPP content increases, the TPP groups aggregate more closely, thereby restricting intramolecular rotation [36,37,38] and resulting in a more pronounced enhancement in performance [15,27,39,40].

The fluorescence lifetime of TPP-MQs was further investigated (shown in Figure 1e,f). The results revealed that an increase in TPP group content within the molecule led to a gradual increase in fluorescence lifetime. The quantum yield is 10.2%, suggesting contributions from both radiative and non-radiative channels. The observed lifetime increase is mainly due to the reduced non-radiative decay caused by restricted intramolecular motions in the rigid matrix, consistent with typical AIE behavior. Multiple models were constructed to fit the experimental data, and an exponential relationship (calculated using Equation (1)) provided the closest match with the underlying trend:

(1) $$\tau =0.434e^{0.118x}+0.890,$$

where *τ* represents the fluorescence lifetime of samples, and the term *x* is designated as the number of TPP groups in the samples. This trend provides the best fit (R^2^ = 0.951) among several models tested.

Equation (1) provides the best empirical fit to the lifetime evolution shown in Figure 1f. Such an exponential dependence can be attributed to a cooperative restriction of intramolecular rotation (RIR) [41,42]. Each additional TPP moiety enhances molecular rigidification within the aggregate in a non-linear manner, leading to a progressive and accelerated suppression of non-radiative decay pathways. This behavior is consistent with reports on nanoparticle and AIE luminogen systems [17,41,42], where the restriction of molecular motion and the local packing density rise cooperatively with chromophore loading. At low TPP content, the lifetime remains nearly unchanged because aggregation is insufficient to induce substantial RIR. Once phase separation becomes prominent, the cooperative packing of TPP units leads to a nonlinear suppression of non-radiative decay, giving rise to the exponential lifetime growth described by Equation (1).

The drastic fluorescence intensity increase observed between 19.3% and 31.7% TPP content (Figure 1c) corresponds to a threshold where phase separation and “sea–island” droplet formation become prominent, effectively concentrating TPP units and restricting rotations. Similar trends are observed in the lifetime data, indicating that both fluorescence intensity and lifetime are governed by the same underlying molecular dynamics. In Figure 1g, the almost negligible emission in 99% H_2_O for polymers with low TPP content reflects insufficient aggregation to trigger AIE. The crossing of curves around three TPP groups signifies a transition point in aggregation behavior, where polymers with different TPP contents begin to adopt similar local packing within droplets, leading to comparable emission characteristics. These analyses demonstrate that the AIE enhancement is closely linked to TPP content, molecular aggregation, and rotational restriction, providing a unified physical interpretation for both intensity and lifetime variations.

Thermal degradation behavior was investigated via thermo-gravimetric analysis (TGA) in Figure 1h, revealing a 5% weight loss temperature above 400 °C. This can be attributed to the exceptional stability of the polysiloxane skeleton [43]. The weight loss is primarily concentrated within the temperature range of 400–600 °C, involving two main processes. Initially, at low temperature, the TPP side groups are eliminated. As the temperature increases, the polysiloxane main chain undergoes fragmentation into smaller molecules through internal cyclization and other mechanisms before being expelled from the system. The final stage is characterized by the presence of only silica, which has a higher melting point, resulting in a residual weight of less than 10%.

The trend in the TGA curve (Figure 1i) also indicates that as the TPP group content increases in the polymer, the proportion of the polysiloxane main chain decreases. Consequently, more TPP groups are eliminated, leading to a reduction in the heat resistance of the polymer. Nevertheless, the thermal stability of TPP-MQs at elevated temperatures remains adequate to meet the stability requirements for practical applications.

**Figure 2:**
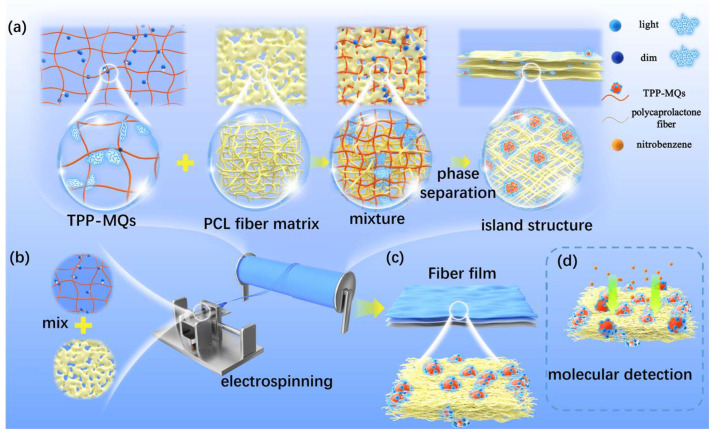
(**a**) Schematic illustration of the phase separation between TPP-MQs and the PCL matrix upon mixing. (**b**) Mixing of the substances and subsequent electrospinning. (**c**) Electrospun fiber membrane and its schematic microstructure. (**d**) Schematic illustration of the molecular detection mechanism on the fiber membrane.

Furthermore, PCL-based fiber films loaded with TPP-MQs micro-particles were fabricated via electrospinning [44], as shown in Figure 2b. Based on molecular dynamics simulations, we propose that driven by compatibility differences, TPP-MQs induce nucleation and growth of the polymer within the PCL fibers, resulting in inter-polymer phase separation and the formation of a sea–island structured polymer membrane [45]. The electrospun membrane forms a three-dimensional mesh structure with a high specific surface area (ranging from tens to hundreds of m^2^/g), significantly enhancing the contact area between target molecules and the TPP-MQs active sites on the fiber surface [46]. The porous network architecture enables rapid penetration of gaseous or liquid-phase target molecules, facilitating their diffusion into the fiber interior for efficient binding with the immobilized TPP-MQs sites, thereby shortening response time and substantially improving detection sensitivity. Within the TPP-MQs/fiber composite system, a key structural transformation occurs due to synergistic compatibility-mismatch-driven and entropy-driven processes: TPP-MQs molecules undergo conformational rearrangement to form spherical microparticles, which exhibit exceptional target molecule capture and fluorescence response efficiency, as shown in Figure 2a,c. This phase separation is driven by the combination of a highly flexible silicon-oxygen backbone and rigid, polar tetraphenylbenzene side groups in the TPP-MQs molecules. To minimize interfacial energy (ΔG), the system evolves toward a spherical geometry, the optimal configuration for reducing the interfacial area per unit volume. Through π-π stacking interactions and the interactions between polar groups, the tetraarylphenyl groups self-assemble into a dense core [47], which aggregates to form a supporting structure. The highly flexible silicon–oxygen backbone extends outward, creating a compliant interface layer that maintains partial compatibility with the polar matrix, thereby forming a flexible outer shell. This results in the formation of spherical “rigid core–flexible shell” particles.

**Figure 3:**
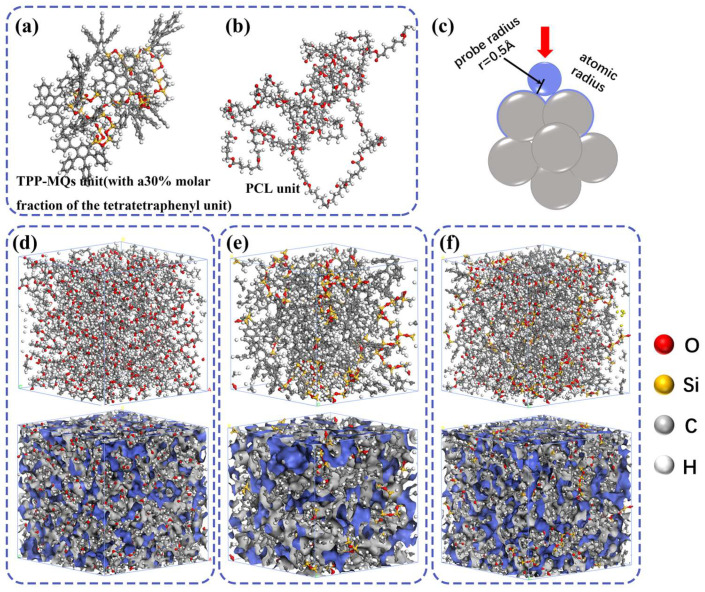
(**a**,**b**) Molecular dynamics simulation units of TPP-MQs and PCL. (**c**) Schematic diagram of the calculation principle of the free volume fraction. (**d**–**f**) Amorphous cells with free volume analysis of PCL, TPP-MQs, and TPP-MQs/PCL, respectively (grey: occupied volume, blue: free volume).

Molecular dynamics simulations confirm the existence of compatibility differences between TPP-MQs and PCL fiber molecules as shown in Figure 3. The results determined the solubility parameters of PCL and TPP-MQs to be 17.6 MPa^1/2^ and 14.4 MPa^1/2^ respectively. For their 1:2 wt% mixture, the composite solubility parameter was calculated to be 16.04 MPa^1/2^. When the solubility parameter difference between two polymers exceeds 2 MPa^1/2^, they exhibit poor compatibility and are prone to thermodynamically driven nucleation and growth-type liquid–liquid phase separation (L-LPS) [48]. Substituting these values into a Flory-Huggins style free-energy expression. According to Flory–Huggins theory, the free energy of mixing is calculated using Equations (2)–(4) [32]. Substituting our data into the Gibbs free energy equation yields a molar free energy of mixing ΔG_M_ ≈ −3.4 J/mol for the modeled composition, which places the system within the spinodal region and predicts spontaneous phase separation rather than kinetically arrested mixing. This approach of combining molecular simulations with thermodynamic theory to demystify phase separation mechanisms is well-established and has been successfully applied to other polymer systems, such as alkaline polymer electrolytes during electrospinning [49].

(2) $$∆G_{M}=∆H_{M}-T∆S_{M},$$

(3) $$\chi_{A}=\frac{V_{A}{\left(\delta_{A}-\delta_{B}\right)}^{2}}{RT},$$

(4) $$∆G_{M}=RT(n_{A}ln\varphi_{A}+n_{B}ln\varphi_{B}+\chi_{A}\varphi_{A}\varphi_{B})$$

ΔG*_M_*, ΔH*_M_*, and ΔS*_M_* represent the molar Gibbs free energy, enthalpy, and entropy of mixing, respectively. *δ_A_* & *δ_B_*, *n_A_* & *n_B_*, *φ_A_* & *φ_B_* denote the solubility parameters, molar quantities, and volume fractions of solvent and solute, respectively. χ*_A_*, *T* and *R* correspond to the Huggins parameter of the solvent, system temperature in Kelvin, and gas constant.

Radial Distribution Function (RDF) analysis (Figure S3) provides further insight into the spatial organization of TPP-MQs within the PCL matrix. In the range of 0–5 Å, the RDF of carbon atoms in the polysiloxane backbone exhibits a pronounced peak, indicating a high local density and strong interaction with PCL segments. This suggests the formation of a stable interfacial layer where the flexible silicane chains are intimately associated with the PCL matrix [50]. Between 5–12.5 Å, the RDF of TPP groups rises significantly, reflecting their aggregation into localized domains. This behavior is attributed to π–π stacking and hydrophobic interactions among the aromatic TPP units, leading to the formation of a rigid, dense core. Notably, at larger radial distances (12.5–20 Å), the RDF profiles of both silicane and TPP components converge and approach a value of 1. This convergence indicates the loss of structural correlation and a transition to a homogeneous molecular distribution at longer length scales. Such behavior is characteristic of amorphous polymer systems, without long-range periodic order [51,52]. The convergence thus supports the presence of microphase-separated “island” structures embedded within a continuous PCL “sea”, consistent with the entropy-driven phase separation mechanism proposed.

**Figure 4:**
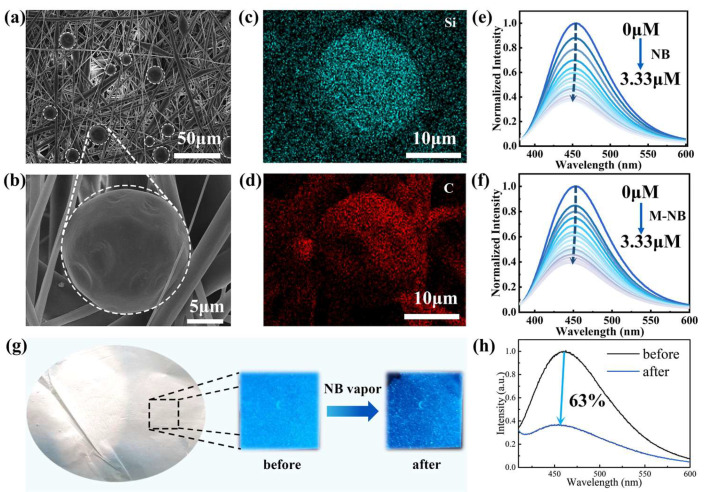
(**a**) SEM image of the electrospun fiber containing TPP-MQs microparticles. (**b**) Enlarged view of the region marked in (**a**). (**c**) EDS elemental mapping of silicon (Si) for the fiber with TPP-MQs microparticles. (**d**) EDS elemental mapping of carbon (C) for the fiber with TPP-MQs microparticles. (**e**,**f**) Fluorescence response of TPP-MQs aggregates upon gradual addition of nitrobenzene (NB) and m-dinitrobenzene (M-DNB) solutions, respectively. (**g**,**h**) Photographs showing the sample and its fluorescence change before and after exposure to NB vapor.

Furthermore, atomic-level trajectory metrics demonstrate the structural reorganization underlying this thermodynamic change. Free-volume analysis of the equilibrated cells shows an increase in heterogeneous free volume upon demixing (Figure 3d–f), and radial distribution functions (RDFs) reveal strong local ordering of TPP aromatic units at short range (5–12.5 Å), consistent with π–π driven core formation. Conformational analyses show that the flexible siloxane segments adopt more extended and varied conformations after domain formation (increased radius of gyration distribution and broader conformational ensemble), which corresponds to an increase in configurational entropy of the siloxane component. Interaction-energy decomposition further indicates that while hetero-contact enthalpy is not favorable, the net free-energy balance becomes negative because the increase in configurational/free-volume entropy compensates the enthalpic cost. This central role of chain conformational entropy in driving and stabilizing phase separation has emerged as a key insight from atomistic simulations of diverse systems, that form membraneless organelles [53]. Taken together, these MD results support the mechanistic picture in which incompatibility (enthalpic mismatch) initiates demixing, but the dominant thermodynamic driving contribution in our modeled composition and conditions arises from an entropy gain associated with chain-conformational rearrangements and increased free volume in the matrix; this combination leads to nucleation and growth of TPP-rich spherical domains embedded in the PCL “sea” (the “sea–island” morphology). Scanning electron microscopy (SEM) observations (Figure 4a,b) confirm the consistency with theoretical predictions: TPP-MQs and PCL exhibit interfacial binding forces at the molecular level. TPP-MQs molecules readily aggregate on the surface of PCL fibers, forming “island-like” spherical particles.

We note the limitations of the model (finite chain lengths, idealized compositions and timescales accessible to MD). Nonetheless, the concordance between solubility-parameter/Flory–Huggins estimates, free-volume and RDF analyses, and the experimentally observed island-in-sea morphology provides mutually consistent evidence for the proposed entropy-assisted phase separation pathway.

This morphology where polymers undergo phase separation to form island-like structures arises from the incompatibility and polar mismatch between the TPP-MQs and the PCL matrix, which contains strongly polar ester groups (-C=O). This mismatch drives the tendency for phase separation. In TPP-MQs, the phenyl side chain, which has poor compatibility with the solvent, forms the inner core of the particle while the silicone–oxygen chain with slightly better compatibility is distributed on the surface. Concurrently, driven by an increase in entropy and the minimization of interfacial energy associated with molecular chain conformation, the TPP-MQs molecules undergo self-assembly, rearranging into an unordered structure and eventually forming spheres embedded within the PCL fibers.

To explore the potential application of the films, the detection capability of TPP-MQs for nitroaromatics was first examined under two different scenarios. Using TPP-MQs with 50% phenyl content as the representative sample, nitrobenzene (NB) and meta-dinitrobenzene (M-DNB) were selected as the nitroaromatic compounds. This pair shares a well-defined photoinduced electron transfer (PET) quenching mechanism with the TPP core but possesses a graduated electron-accepting capability. The comparison between them allows for a sensitive evaluation of how the sea–island fibrous architecture modulates analyte accessibility and the resulting quenching efficiency. In previous studies, we have fully established their quenching behavior for TPP-based polysiloxanes, which enabled us to focus on evaluating the impact of the newly formed sea–island fibrous structure on the sensing performance [28]. Upon adding a nitrobenzene solution to TPP-MQs aggregates (dispersed in a poor solvent), a significant decrease in fluorescence intensity was observed. At a concentration of 3.33 µM, the TPP-MQs aggregates exhibited more than a 50% reduction in fluorescence intensity compared to the initial value, as shown in Figure 4e,f. Moreover, the film was exposed to nitrobenzene vapor for a specific duration to monitor fluorescence variation. As shown in Figure 2d and Figure 4g,h, brief exposure of the polymer film to nitrobenzene vapor resulted in a marked decrease in fluorescence intensity, demonstrating a clear and sensitive fluorescence response toward nitroaromatic analytes [54].

Given the strong electron-withdrawing nature of nitrobenzene, the observed fluorescence quenching is mainly attributed to the formation of ground-state charge-transfer complexes (static quenching) between NB molecules and the electron-rich TPP units embedded in the aggregated domains. Such complexation has been systematically verified in our previous study on structurally analogous polysiloxane-based AIE systems and does not require remeasurement of absorption or lifetime changes [28]. Owing to the static yet reversible nature of the quenching pathway, the binding process is non-destructive under mild conditions. The fluorescence signal can be fully recovered upon the removal of NB molecules from the TPP cores, which is consistent with the reversible complexation mechanism we have established. This explains the restoration of the original fluorescence intensity after NB exposure.

## 4. Conclusions

In summary, TPP groups with intrinsic AIE properties were successfully incorporated into polysiloxane backbones to yield fluorescent TPP-MQs. When processed into fibrous films via electrospinning, the intrinsic incompatibility of the polymer matrix induced entropy-driven phase separation, generating a distinctive “sea–island” morphology. This hierarchical porous structure not only enlarged the specific surface area but also facilitated analyte diffusion, thereby overcoming the fluorescence quenching commonly observed in conventional nanostructures. Molecular dynamics simulations further corroborated the entropy-governed mechanism of phase separation and provided direct insight into the conformational rearrangements of TPP-MQs within the fibrous matrix. Benefiting from this architecture, the obtained films exhibited efficient adsorption capacity and highly sensitive fluorescence response toward target analytes. Overall, this work establishes a feasible and generalizable strategy for translating the luminescent properties of AIE gens into stable, high-performance sensing platforms.

## Data Availability

The original contributions presented in this study are included in the article/Supplementary Material. Further inquiries can be directed to the corresponding author(s).

## Supplementary Materials

The following supporting information can be downloaded at: https://www.mdpi.com/article/10.3390/polym17243252/s1, Figure S1: Infrared spectra of (a) tetraphenylbenzene (TPP-MQs) silicone oil and (b) vinyl silicone oil; Figure S2: ^1^H NMR of TPP-VMQs; Figure S3: The radial distribution function of carbon atoms between the TPP or silicane component and PCL in the PCL/TPP-MQs system; Figure S4: Stern-Volmer plots of TPP-MQs in H2O/THF (99:1 *v*/*v*) solution toward different nitroaromatic compounds (Linear range: 0-3.33µM); Table S1: The amount of raw material used in sample preparation; Table S2: Basic properties of TPP-MQs; Table S3: LOD calculation.
