# Supplementary material for: Entropy-Driven Phase Separation of AIE Polysiloxanes into Porous Fibrous Films for Fluorescence Sensing
Source: Polymers (Basel). 2025 Dec 6;17(24):3252. doi: 10.3390/polym17243252 (PMC12736471; doi:10.3390/polym17243252)
Supplement: Supplementary file 1 [file polymers-17-03252-s001.zip › polymers-3980414-supplementary.pdf]

Supporting Information

Entropy-Driven Phase Separation of AIE Polysiloxanes into Porous Fibrous Films for Fluorescence Sensing

Jingxuan Zhu[a,b,c,], RuiRui Shi[a], Yifan Wang[a], Yan Chen[a], Yan Liang[d*], Hua Wang[a,b,c,e*] and Chuanjian Zhou[a,b,c,e]

[a] *School of Materials Science and Engineering, Shandong University, Jinan 250061, P. R. China*

[b] *State Key Laboratory of Coatings for Advanced Equipment, Jinan 250061, P. R. China*

[c] *Key Laboratory of Special Functional Aggregated Materials, Ministry of Education, Jinan 250100, P. R. China*

[d] *School of Food Science and Technology, Qilu University of Technology, Jinan250353, China*

[e] *Shandong University-Weihai Research Institute of Industrial Technology, Weihai 264209*

*Corresponding authors:

hwhwang@sdu.edu.cn (Hua Wang)

liangyan@qlu.edu.cn (Yan Liang)

Table S1 The amount of raw material used in sample preparation.

The amount of raw material	MVQ / g	TPCP / g
3.2TPP@PDMS	8.01	1.97
6.6TPP@PDMS	8.03	3.87
10.6TPP@PDMS	8.04	7.75
19.3TPP@PDMS	8.00	11.14
31.7TPP@PDMS	8.05	15.28

Note: xxTPP represents the molar content of TPP units in TPP-MQs.

Table S2 Basic properties of TPP-MQs

Samples	TPP content/%	Mη/(g/mol)	Phase state
3.2TPP@PDMS	3.20	4307	l
6.6TPP@PDMS	6.60	4241	l
10.6TPP@PDMS	10.59	5245	l
19.3TPP@PDMS	19.28	6088	s
31.7TPP@PDMS	31.65	6889	s

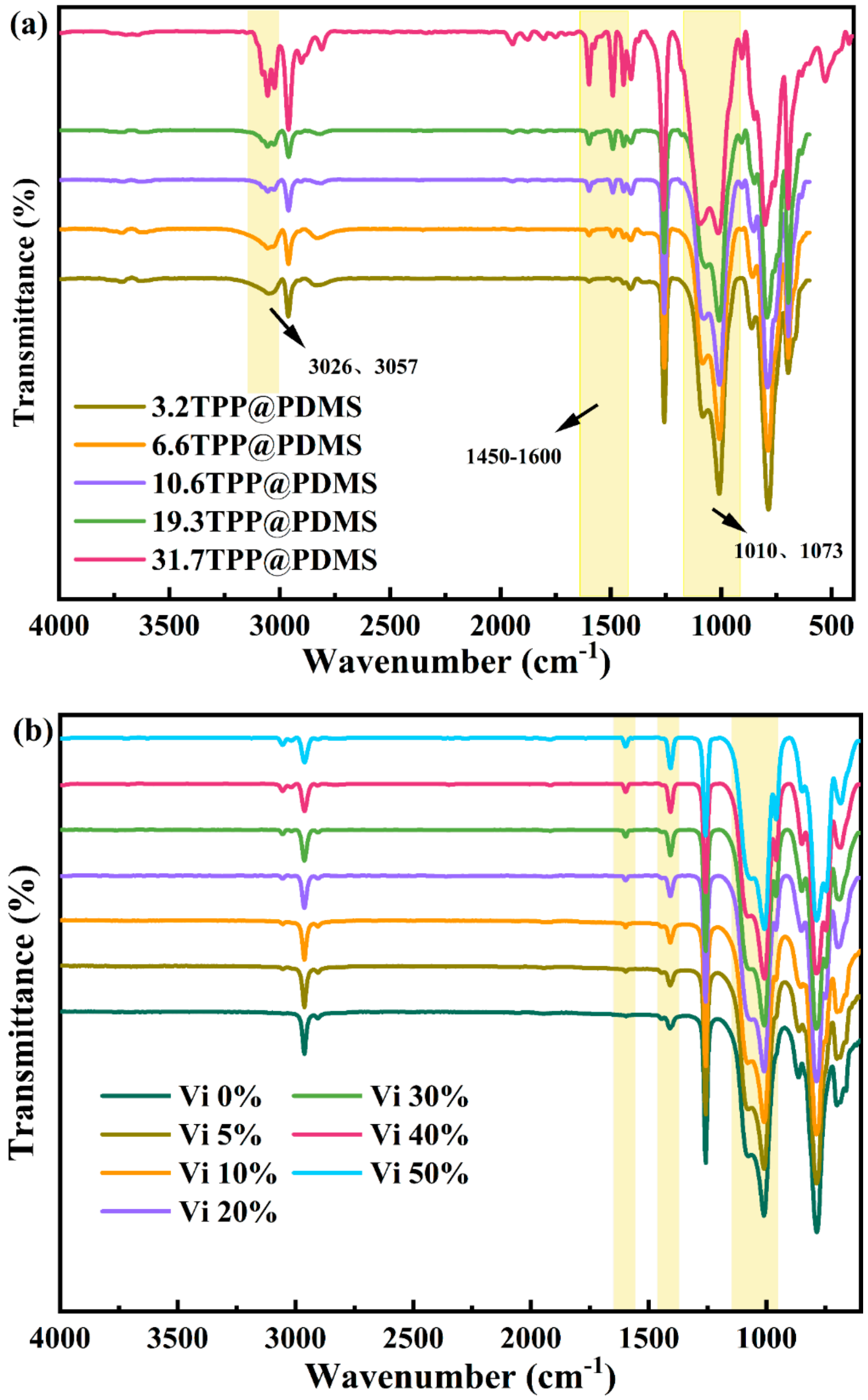

Figure S1. Infrared spectra of (a) tetraphenylbenzene (TPP-MQs) silicone oil and (b) vinyl silicone oil.

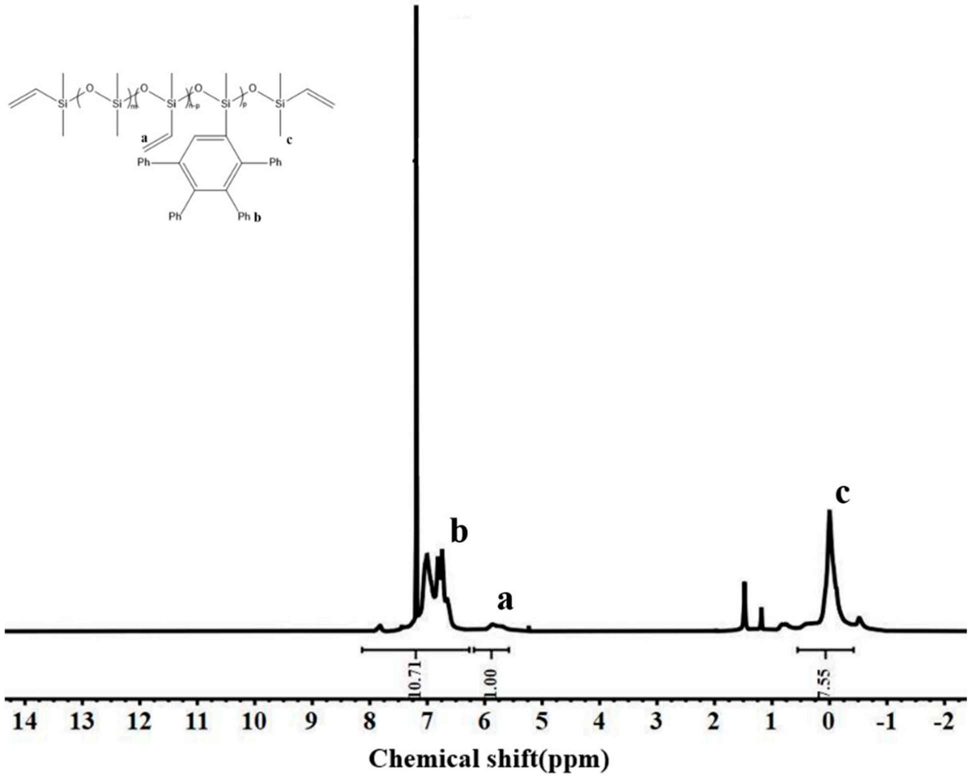

Figure S2. ^{1}H NMR of TPP-VMQs

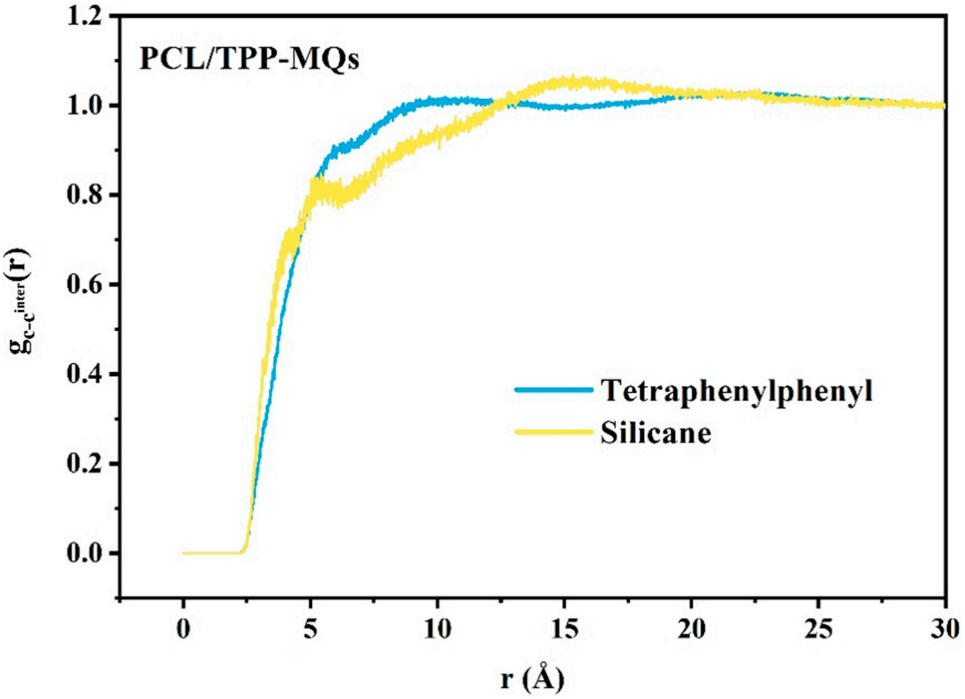

Figure S3. The radial distribution function of carbon atoms between the TPP or Silicane component and PCL in the PCL/TPP-MQs system

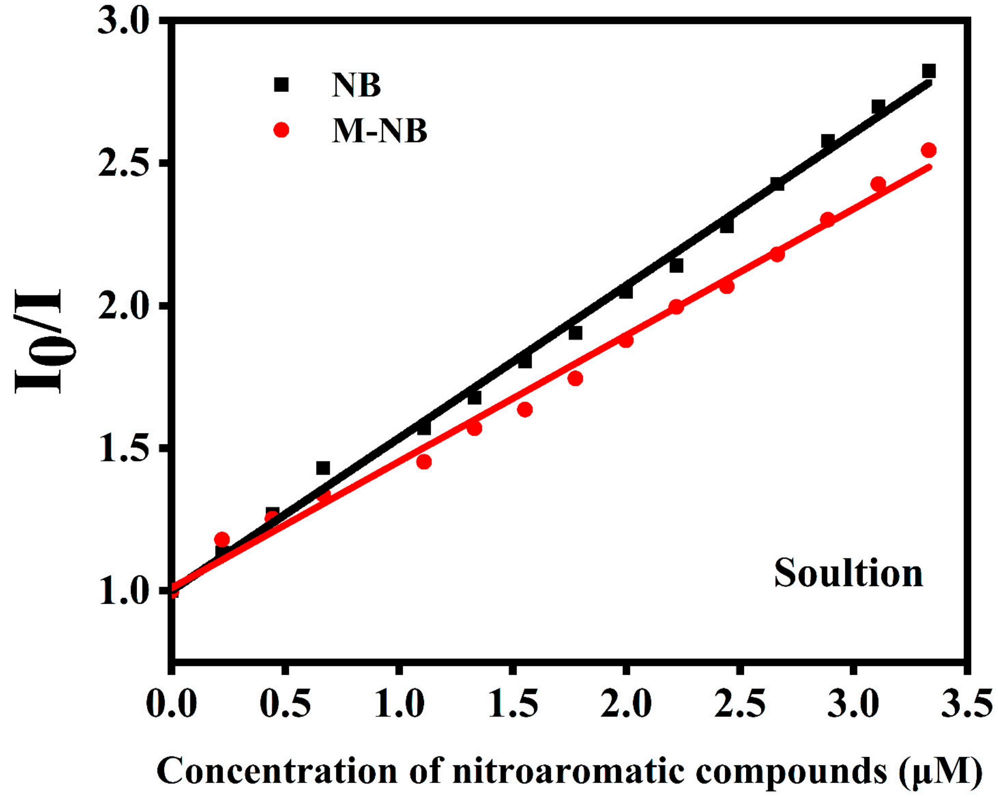

Figure S4. Stern-Volmer plots of TPP-MQs in H_2O/THF (99:1 v/v) solution toward different nitroaromatic compounds (Linear range: 0-3.33μM)

Table S3 LOD calculation

Analyte	Ksv (M^{-1})	σ	LoD(nM)
NB	5.35×10^5	0.00296	16.6
m-NB	4.43×10^5	0.00296	20.0

Note: LoD = 3σ/Ksv
